# The Duties of Examiners in Lunacy

**Published:** 1913-09

**Authors:** Carey D. Davie

**Affiliations:** Surrogate of Cattaraugus County, Salamanca


### The Duties of Examiners in Lunacy

BY CAREY D. DAVIE
Surrogate of Cattaraugus County, Salamanca

DURING the benighted conditions of the Middle Ages, when the empire of the human intellect was limited, and the creations of distorted fancies accepted as facts, the insane were regarded as victims of evil spirits, struggling under the displeasure of an omnipotent power. At times they were treated as outcasts and a menace to society. At other times regarded with mysterious veneration as beings under the special protection of an over-ruling providence. The alleviation of their afflictions was outside the realms of medical skill; their comforts and safety to no extent the subject of legislative action or judicial concern; their recovery depended entirely upon unassisted natural causes; their physical protection merely a matter of accidental environments; but, when the shadows of that superstitious period were dispelled by the light of intelligent investigation, this unfortunate affliction of humanity became the subject of thoughtful observation, and, during recent years, some of the brightest intellects have been industriously employed in the study of the care, treatment and welfare of the insane. In every civilized country the greatest solicitude is now manifested for the comfort and protection of this unfortunately afflicted class. Public sentiment has undergone important modifications; common sense has superceded prejudice, and intelligent methods have displaced superstitious beliefs and customs. Not long ago the so-called lunatic asylums were regarded with horror, and not entirely without reason, for they were merely shambles or herding places for those deprived of reason, where the sole element of control was physical force. These institutions are now replaced, in name and in fact, by the State Hospitals for the Insane, presided over by physicians and alienists of experience and ability, institutions built, equipped and maintained at public expense, and from which none are excluded in consequence of poverty; where the first effort in every case is to effect a radical cure, if possible, and, failing in that, to ameliorate the conditions and minimize the effect of the mental disorder upon the comfort and duration of the patient's life.

In the treatment and custody of the insane, the hearty co-operation of the medical and legal professions is demanded, but the functions of these two professions radically differ. It is the province of the physician to correctly diagnose the disorder, determine its cause, carefully investigate the antecedents, which throw light upon the general pathology, and eradicate, not only the abnormal mental condition, but the exciting cause. With these matters, the lawyers and courts have no concern. It is well said in Bucknill's Treatise on "Insanity and its Legal Relations," that "To the lawyer it matters not *how* the seed of insanity is sown, nor the growth of the plant, except as confirmatory evidence that the plant is there; with him the sole question is its existence, its degree and its influence upon the conduct, and is, therefore, a moral and not a medical consideration." It is the results of the research and investigation of the medical profession which afford a basis of action for judicial determination. It is impossible for the law to reach just and proper results without the intelligent information furnished by the physician. The physician lays the foundation upon which the courts erect the superstructure; the physician establishes the premises from which the law reasons and reaches its conclusions. Consequently, the responsibility of the medical expert is two fold; first, to his patient in securing the best possible results of his treatment, and, second, to the courts in furnishing such professional information as will lead to proper adjudication regarding the custody, liberty and property rights of the patient, and his relations to society, civilly and criminally.

Under the provisions of the Insanity Law of this State, the evidence upon which the committing magistrate acts is the certified expression of opinion of examiners in lunacy. In most cases, such certificate is the only proof of the mental status of the party whose commitment is sought; while the law now requires that notice be served upon the patient of an application for his commitment, yet it is very rarely the case that any one appears in his behalf. Such being the case, the examiner should appreciate the supreme importance of his functions in this connection; he has to deal with human liberty. He stands as a sentinel at the approach to our Insane Hospitals, admitting those whose mental condition requires skilled treatment and observation, and turning those back who are offered merely as a sacrifice to improper motives. In the performance of his duties he should never overlook the blacker side of human nature, nor underestimate the force of motives springing from selfishness, avarice, illicit relations and affections, restlessness and impatience under the restraint imposed by the rules of society and common decency. He should always have in mind that there are a multitude of reasons for seeking the incarceration of persons within the walls of the hospital other than honest desire to benefit and protect them.

An instance has come under my observation where a husband finding his wife to be an inconvenient incumbrance seizes upon a slight and absolutely harmless delusion on her part as a pretext for securing her commitment. The local poor authorities, in their zeal to protect their municipalities from financial liability for the maintenance of harmless and inoffensive paupers, sometimes find it politic to transfer responsibility to the State by securing their commitment to the State institution. The examiner should always be on his guard against attempts to make use of him in his official capacity to secure a commitment where the same is absolutely unjustified. Such a preceeding is an outrage upon the patient and may prove disastrous to the examiner. The difficulties the examiner encounters are often serious and perplexing. There are occasionally instances where the manifestations of mental disorder are so marked, violent and uncontrollable as to preclude the possibility of a mistaken diagnosis but in many cases of the most dangerous types of insanity, the patient is quiet under observation, cunning and crafty in disguising his symptoms, skilled in deceit and dissimulation, requiring the greatest patience and ingenuity on the part of the examiner to develop and bring to light the latent fires of wrecked mentality. The objective symptoms of disturbed mentality are as varied as the hues of the kaleidoscope. When the physician begins investigation in the hazy realms of mental disorders, the most thoughtful good judgment is required to guard against erroneous conclusions. No two cases are alike. Each case presents its own marked peculiarity. In one instance he discovers the existence of hallucinations, where, as described by Taylor, "The patient experiences sensations which are supposed by him to be produced by external impressions, although no material object acts upon the senses at the time." An illustration of this condition was recently presented in a contested will case tried before the writer. The testatrix was an old lady who entertained and strenuously adherred to the belief that a circus with all its varied paraphernalia was encamped in front of her residence; she heard the music of the band, laughed at the antics of the clown, admired the elephants and tigers, and insisted that the paper on the walls of her room was flaming circus posters. In all other particulars she was rational; she had an intelligent understanding of her business affairs and it was held that she possessed testamentary capacity. In such a cause, the examiner in lunacy would hardly have been justified in certifying that the patient should be committed. In another case the testatrix had become possessed of the delusion that she was a burden to her family, and in consequence began considering self-destruction. She made inquiries as to the amount of arsenic required to take one's life and shortly thereafter was found dead, having committed suicide by hanging from a rafter in her barn. Here a timely examination and commitment would have averted the calamity and possibly have eventually lead to a recovery. In another case a man who had for some time been afflicted with arterio-sclerosis made his will and shortly thereafter threw himself in front of an approaching railroad train and was crushed to death. In the investigation of this case, the interesting question arose as to what extent the fact of suicide was evidence of insanity, and an experienced alienist, called as a witness, expressed the opinion that self-destruction was not in and of itself absolute proof of insanity, citing instances where persons perfectly sane had taken their lives and deliberately and intelligently planned so to do. Again the examiner will encounter the manifestations of delirium, incoherent speech, extreme watchfulness, purposeless action, inability to fix attention and concentrate the thoughts, changing hallucinations, delusions and illusions. Again he will observe a patient with that loss of expression and marked vacuity which indicates the existence of dementia, or he encounters those deplorable conditions described by Kirchoff as "Depression marked by a feeling of misery in excess of what is justified by the circumstances in which the individual is placed," and denominated melancholia. Again he observes the manifestation of extreme hilarity and exhultation where the patient entertains the belief that he possesses great wealth, power and social influence, is perfectly happy and contented, never suicidal in his tendencies, but homicidal in his designs against those who interfere with his plans and ambitions. Again his attention is called to the fact that a merchant is annoyed by the operations of a kleptomaniac. A community is terrorized by the acts of a pyromaniac, one afflicted with corpolalia shocks his associates with his obscenity and profanity; the conduct of the victim of morphiomania, or dipsomania, humiliates his friends and family. The subject of nymphomania becomes a social outcast; or the examiner is startled at the fiendish ingenuity of the crime of some paranoiac. In judicial procedure, where the question of mental responsibility is at issue he encounters the defence of acute delirious mania, religious or erotic paranoia, hypochondriac, epileptic, hysterical, temporary or emotional insanity and numerous other form of mental aberration, chronic or acute. In one famous murder trial, after the jury had had the case under consideration for a considerable time and were unable to agree, it returned to court and asked for instructions. "Your Honor," said the foreman, "we are all agreed that the defendant was entirely sane immediately before the shooting and that he was entirely sane immediately after, but we are unable to agree upon the question of his sanity at the precise instant that he fired the shot." Here the defense was temporary insanity and experts had testified as to the mental condition of the defendant and created a situation where the court in answer to the juror's inquiry was obliged to say: "Gentlemen of the Jury, it is incumbent upon the prosecution to convince you beyond a reasonable doubt as to the defendant's sanity at the time of committing the act. If a reasonable doubt exists in your minds in that particular the defendant is entitled to the benefit of such doubt." The jury retired and very soon returned with a verdict of "Not Guilty."

I have thus referred to a considerable extent to the difficulties which the medical examiner encounters for the purpose of substantiating my contention that no physician, however extensive his general practice, should assume to act as an examiner in lunacy without possessing special and technical qualifications along that line.

In some respects our insanity law is lax, insufficient and dangerous. The protection of personal liberty was regarded by the framers of the Constitution as of sufficient importance to demand a special guaranty. In our criminal jurisprudence one charged with crime, where the question of mental responsibility is involved, has the protection of every legal presumption and can be convicted and incarcerated only upon the unanimous verdict of twelve men. The alleged incompetent person cannot be deprived of the control of his property except through the instrumentality of proceedings instituted for that purpose, the filing a petition, appointment of a commission, summoning a jury of not less than twelve or more than twenty disinterested persons, taking of evidence, a finding of incompetency signed by at least twelve of such jurors, yet under the provisions of the insanity law one may be deprived of his liberty simply upon the certified opinion of two examiners in lunacy, probably qualified and honest but possibly not, approved of by a judge who may regard his functions in the premises as purely formal and who relies entirely upon such certificate. The apology is sometimes made for a careless and superficial examination of the mental condition of the patient that no harm can ensue because the alienist in charge of the institution to which the commitment is made will detect the fact if no insanity exists and refuse to accept the patient, but such an excuse is neither professional nor complimentary to the examiner, nor one affording protection in the forum of the examiner's honest conscience, nor in a court of law when he is sued for false imprisonment. The experts in charge of our larger institutions can hardly be expected to immediately examine every incoming patient and at once determine the question of his sanity. It often requires several days' observation to ascertain the actual mental condition of a patient and the authorities in charge of the institution have the right to and necessarily do rely upon the presumption that the examiners, in making their certificate, are properly qualified and that they have carefully performed their duties. The statute permits any physician of three years' standing to legally qualify as an examiner in lunacy even if he has never had an insane patient and is absolutely unable to differentiate a case of secondary dementia from one of a harmless hallucination. The statute should be amended in such a manner as to require a reasonable amount of experience in the observation of mental disorders before a physician may be permitted to qualify as an examiner in lunacy, and the method of procedure in securing the commitment of a patient should be more thoroughly safeguarded; at least the same degree of care should be required in a proceeding to deprive one of his liberty as of the custody of his property. The statute will be the subject of criticism so long as its provisions render it possible to secure the incarceration of an individual who may be entirely sane, simply upon the certificate of two dishonest or incompetent examiners and the order of a careless committing magistrate.

After making the examination and determining the patient's condition, the next duty of the examiner is to prepare the proper certificate. This should be carefully done. Many certificates are so carelessly prepared as to be absolutely farcical. The statute requires that the examiner in addition to a statement of his opinion regarding the sanity of the patient, should also set forth the facts upon which such opinion is based. I recall an instance where the only fact recited in the certificate was that the patient, a woman, had "called her husband a scoundrel and a Democrat"—a very unlikely combination. In another case the fact was very carefully recorded that "she said nothing during our examination"—hardly satisfactory evidence for judicial action. Again it was recited that "the patient acted queer." If every person who sometimes acts queerly is a subject for the insane hospital, we might not find any committing magistrate or examiner at large. These conditions are largely the result of absolute carelessness in the preparation of the certificate. They indicate a belief on part of the one certifying "that anything is good enough for a lunatic." They show want of appreciation on the part of the examiner of the serious importance of the duties they are performing. The statute requires that sufficient facts be stated in the certificate to enable the committing magistrate to verify the opinion of the examiner. A compliance with these requirements is jurisdictional and a certificate which does not comply with the statute affords no protection to either the examiner or the judge when the legality of the commitment is challenged. It is not a comfortable situation for the examiner to be called upon to defend himself before a jury against a claim for large damages in an action for false imprisonment.

These suggestions are made, not in any sense of pedantry, but simply as a matter of caution to the examiner and magistrate in the performance of a most important duty.

---

SUBSEQUENT HISTORY OF 1000 PATIENTS WHO RECEIVED TUBERCULIN TESTS. Johanna Gelien and Louis Hamman, *Johns Hopkins Hosp. Bull.*, June, 1913.

Table 1—The present condition of 632 patients classified from three to four years before:

| | Sought | Reports | Well | Not improved | Became Tuberculous | | Death not from tuberculosis |
|---|---|---|---|---|---|---|---|
| | | | | | Living | Dead | |
| Not tuberc.. | 188 | 110 | 72—65% | 31—28% | 3— 2.7% | 1—0.9% | 3—2.7% |
| Doubtful........ | 429 | 258 | 176—68 | 57—22 | 7— 2.7 | 9—3.5 | 9—3.5 |
| Probable........ | 78 | 47 | 29—61 | .................. | 11—23 | 7—15 | .................. |
| Tuberculous .. | | | | | | | |
| Stage I....... | 35 | 21 | 13—62 | 4—19 | ................ | 3—14 | 1—4.7 |
| Stage II...... | 79 | 47 | 15—32 | 10—21 | ................ | 22—47 | .................. |
| Stage III.. . | 191 | 149 | 11— 7 | 10— 6.7 | ................ | 128—86 | .................. |

Table 2—The after history of patients in relation to the Tuberculin tests:

| | | Became tuberculous | Did not become tuberculous |
|---|---|---|---|
| Not tuberculous | Reacted to 1% conjunctival test......... | 0— 0 % | 1—100% |
| | Reacted to 5% conjunctival test......... | 0— 0 | 5—100 |
| | Reacted to the cutaneous test............ | 1— 3 | 31— 97 |
| | Negative to conjunctival test............ | 4— 3.8 | 100— 96 |
| | Negative to cutaneous test................ | 3— 3.8 | 75— 96 |
| Doubtful | Reacted to 1% conjunctival test.. .... | 4—11 | 33— 89 |
| | Reacted to 5% conjunctival test........ | 3— 7 | 41— 93 |
| | Reacted to the cutaneous test.......... | 9— 6.7 | 125— 93 |
| | Negative to conjunctival test........... | 9— 5 | 168— 95 |
| | Negative to cutaneous test............... | 7— 5.6 | 117— 94 |
| Probable | Reacted to 1% conjunctival test......... | 11—69 | 5— 31 |
| | Reacted to 5% conjunctival test......... | 5—26 | 14— 74 |
| | Reacted to the cutaneous test............ | 17—46 | 20— 54 |
| | Negative to conjunctival test ........... | 2—17 | 10— 83 |
| | Negative to cutaneous test................. | 1—10 | 9— 90 |

